# Glutathione and mitochondria

**DOI:** 10.3389/fphar.2014.00151

**Published:** 2014-07-01

**Authors:** Vicent Ribas, Carmen García-Ruiz, José C. Fernández-Checa

**Affiliations:** ^1^Department of Cell Death and Proliferation, Institute of Biomedical Research of Barcelona, Consejo Superior de Investigaciones Científicas (IIBB-CSIC)Barcelona, Spain; ^2^Liver Unit, Hospital Clínic, Centre Esther Koplowitz, Institut d’Investigacions Biomèdiques August Pi i Sunyer (IDIBAPS)–Centro de Investigación Biomédica en Red de Enfermedades Hepáticas y Digestivas (CIBERehd)Barcelona, Spain; ^3^Research Center for Alcoholic Liver and Pancreatic Diseases and Cirrhosis, Keck School of Medicine, University of Southern CaliforniaLos Angeles, CA, USA

**Keywords:** glutathione, mitochondria, cholesterol, reactive oxygen species, steatohepatitis, Alzheimer disease

## Abstract

Glutathione (GSH) is the main non-protein thiol in cells whose functions are dependent on the redox-active thiol of its cysteine moiety that serves as a cofactor for a number of antioxidant and detoxifying enzymes. While synthesized exclusively in the cytosol from its constituent amino acids, GSH is distributed in different compartments, including mitochondria where its concentration in the matrix equals that of the cytosol. This feature and its negative charge at physiological pH imply the existence of specific carriers to import GSH from the cytosol to the mitochondrial matrix, where it plays a key role in defense against respiration-induced reactive oxygen species and in the detoxification of lipid hydroperoxides and electrophiles. Moreover, as mitochondria play a central strategic role in the activation and mode of cell death, mitochondrial GSH has been shown to critically regulate the level of sensitization to secondary hits that induce mitochondrial membrane permeabilization and release of proteins confined in the intermembrane space that once in the cytosol engage the molecular machinery of cell death. In this review, we summarize recent data on the regulation of mitochondrial GSH and its role in cell death and prevalent human diseases, such as cancer, fatty liver disease, and Alzheimer’s disease.

## INTRODUCTION

**FIGURE 1:**
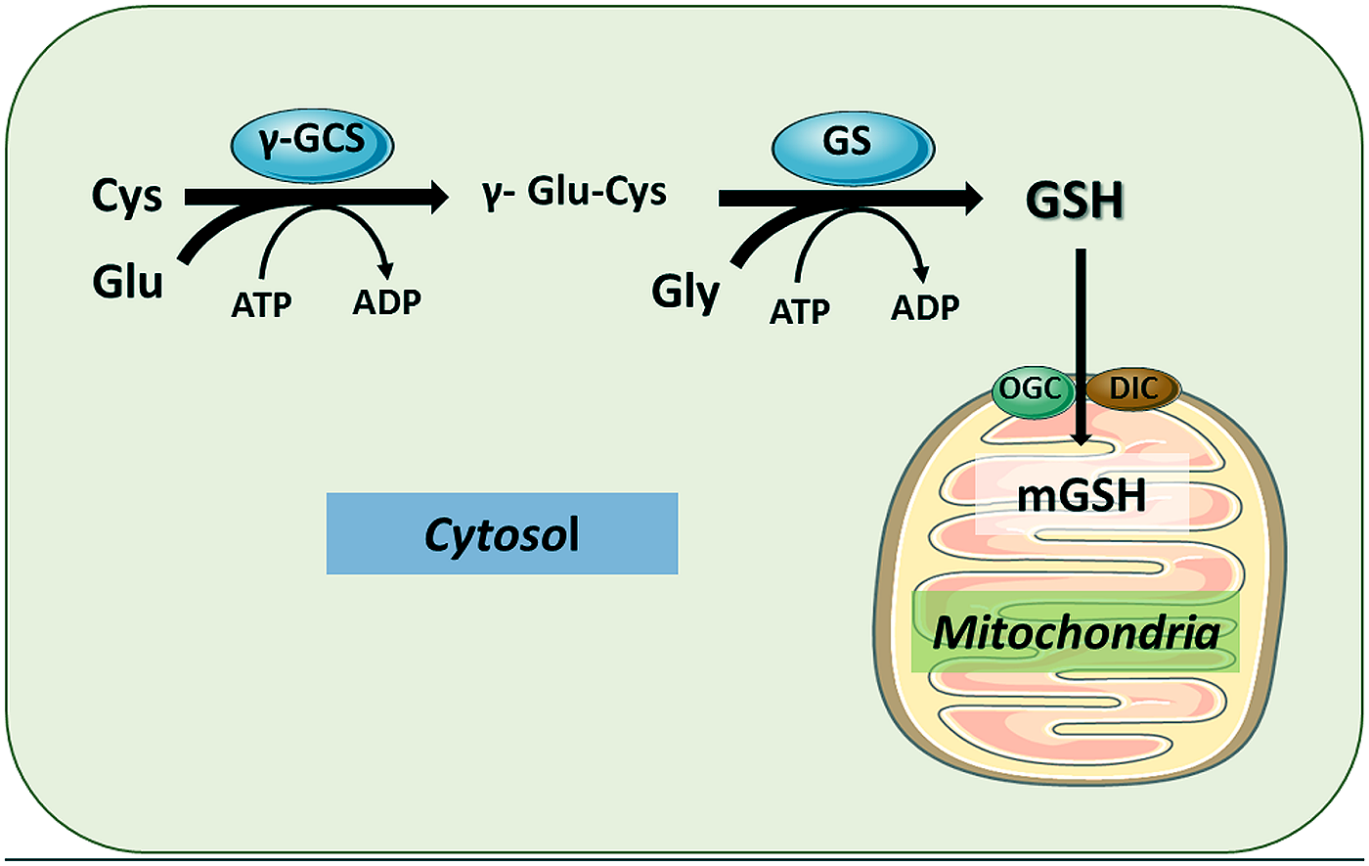
**Glutathione synthesis in cytosol and compartimentalization in mitochondria.** GSH is synthesized from its constituent amino acids in the cytosol by the sequential action of γ-glutamylcysteine synthase (γ-GCS) and GSH synthase (GS). The functions of GSH are determined largely by the –SH of cysteine as by its role as a cofactor for antioxidant enzymes. Once synthesized in the cytosol, GSH can be transported to mitochondrial matrix by different carriers, particularly the 2-oxoglutarate carrier (OGC) and the dicarboxylate carrier (DIC), located in the mitochondrial inner membrane. The function the OGC has been shown to be dependent on changes in mitochondrial membrane dynamics.

Glutathione (GSH), the major intracellular thiol compound, is a ubiquitous tripeptide produced by most mammalian cells and it is the main mechanism of antioxidant defense against reactive oxygen species (ROS) and electrophiles. GSH (γ-glutamyl-cysteinyl-glycine) is synthesized *de novo* in two sequential enzymatic ATP-dependent reactions. In the first step, cysteine and glutamate are linked in a reaction catalyzed by the γ-glutamylcysteine synthase (γ-GCS) to form γ-glutamylcysteine. This first reaction is the rate-limiting step in the synthesis of GSH and is regulated by cysteine availability. The completion of GSH synthesis is catalyzed by glutathione synthetase (GS), in a reaction in which γ-glutamyl-cysteine is covalently linked to glycine (**Figure 1**). The antioxidant function of GSH is determined by the redox-active thiol (-SH) of cysteine that becomes oxidized when GSH reduces target molecules (Pompella et al., 2003). Upon reaction with ROS or electrophiles, GSH becomes oxidized to GSSG, which can be reduced to GSH by the GSSG reductase (GR). Thus, the GSH/GSSG ratio reflects the oxidative state and can interact with redox couples to maintain appropriate redox balance in the cell.

The synthesis of GSH from its constituent amino acids occurs exclusively in cytosol, where γ-GCS and GS reside. However, GSH is found in intracellular organelles including endoplasmic reticulum (ER), nucleus, and mitochondria to control compartment-specific needs and functions (Mari et al., 2009, 2010). Except for the ER, intracellular GSH is mainly found in its reduced form. While the percentage of the total cell GSH content found in mitochondria is minor (10–15%), the mitochondrial glutathione (mGSH) concentration is similar to that found in the cytosol. As GSH has a net negative charge at physiological pH, the high concentration of mGSH implies the existence of specific transport systems that work against an electrochemical gradient (Griffith and Meister, 1985; Garcia-Ruiz et al., 1994; Mari et al., 2009, 2010). As discussed below, despite being a small fraction of total intracellular GSH, mGSH plays a critical function in the maintenance of mitochondrial function and cell survival (Lash, 2006; Mari et al., 2013).

Mitochondria in mammalian cells generate most of the cellular energy by means of the oxidative phosphorylation (OXPHOS) that is essential for myriad cellular functions. OXPHOS provides an efficient mechanism to couple electron transport to synthesize ATP from ADP. Mitochondria are also involved in key cellular functions such as Ca^2^^+^ homeostasis, heme biosynthesis, nutrient metabolism (Cheng and Ristow, 2013), steroid hormone biosynthesis, removal of ammonia, integration of metabolic and signaling pathways for cell death and autophagy (Hammerman et al., 2004; Renault and Chipuk, 2013). Emerging evidence indicates a central role of mitochondria in initiating signals in response to metabolic and genetic stress which affects nuclear gene expression, causing changes in cell function (Raimundo, 2014). Mitochondria contain multiple copies of their own genome, mitochondrial DNA (mtDNA), which encodes for 13 polypeptides of the OXPHOS and respiratory chain, as well as two ribosomal RNAs and 22 transfer RNAs necessary for translation of polypeptides inside mitochondria. As a consequence, the main mitochondrial proteome (~1500 proteins) is encoded by the nucleus, translated in the cytosol and imported into the mitochondria through specific translocator complexes (TIM and TOM) of the inner mitochondrial membrane (IMM) and outer mitochondrial membrane (OMM), respectively.

Oxidative phosphorylation is organized in a series of subsequent steps involving several redox centers distributed in five protein complexes embedded in the IMM (Sun et al., 2013; Venditti et al., 2013). Complex I obtain the electrons from NADH (NADH-coenzyme Q oxidoreductase) and complex II (succinate-coQ oxidoreductase) from succinate. Both these two complexes, independently of each other, use the lipid soluble carrier located into the IMM, ubiquinone (coenzyme Q) to form ubiquinol. From ubiquinol, the electrons pass down the redox gradient through complex III (coenzyme Q-cytochrome *c* oxidoreductase) to cytochrome *c*, then to complex IV (cytochrome *c* oxidase) and to the final acceptor, oxygen (O_2_) to produce water. The fall in electron potential energy through this electron transport chain (ETC) is used to pump protons out the mitochondrial matrix to the intermembrane space (IMS). This proton pumping creates a proton-motive force consisting of electrical and proton gradients. This force is used by the fifth protein complex (Complex V, ATP synthase) to regenerate ATP from ADP. The proton-motive force created by the ETC is also used for many additional mitochondrial processes, especially those related with transport across the IMM (Kulawiak et al., 2013).

Although the primary function of mitochondria is to generate ATP as an energy molecule required for countless cell functions, a small fraction of electrons from the ETC are transferred directly to O_2_, resulting in the generation of the superoxide anion, which can give rise to other ROS as well as reactive nitrogen species (RNS). Mitochondria are the primary intracellular site of oxygen consumption and the major source of ROS, most of them originating from the ETC. In accordance with this, it has been estimated that the steady-state concentration of superoxide in the mitochondrial matrix is 5- to 10- fold higher than in the cytosol (Cadenas and Davies, 2000). Associated with this constant flow of ROS generation, mitochondria are also a target for the damaging effects of oxygen radicals (Fernandez-Checa and Kaplowitz, 2005; Kaelin, 2005; Orrenius et al., 2007).

Although ROS generated under physiological conditions are not harmful, and likely play a signaling role, toxic or pathological conditions that lead to an impairment of mitochondrial function can increase the release of ROS. Mitochondrial ROS are increased under hypoxia, ischemia/reperfusion injury, chemical stress, drug treatment, and under many pathophysiological conditions (Srinivasan and Avadhani, 2012). Despite that mitochondria are exposed to the generation of oxidant species, the existence of an efficient antioxidant defense system, of which mGSH is a critical component, prevents or repairs oxidative damage generated during normal aerobic metabolism (Mari et al., 2013). In the following sections, we summarize some of the most important aspects of mGSH physiology, its role in mitochondrial function and release of mitochondrial apoptotic factors and the impact of its depletion in disease.

## MITOCHONDRIAL ROS GENERATION AND DEFENSE

**FIGURE 2:**
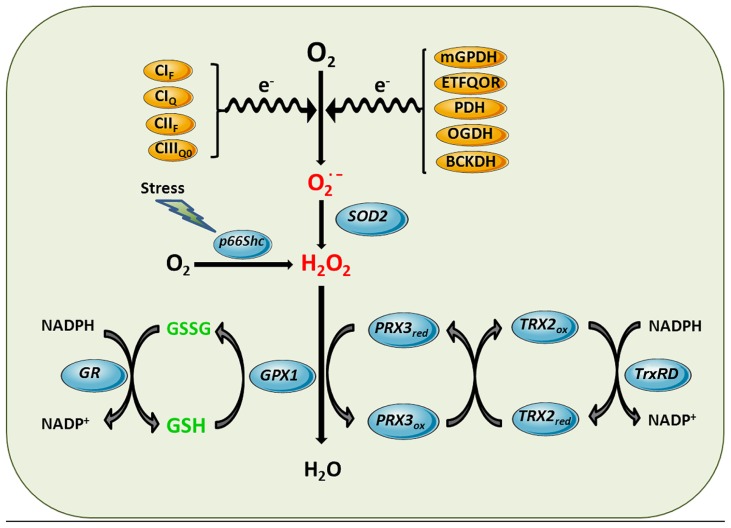
**Mitochondrial ROS generation and antioxidant defense systems.** Complex I flavin site (CI_F_), Complex I ubiquinone site (CI_Q_), Complex II flavin site (CII_F_), and Complex III_Qo_ (CIII_Q0_) are sites of the ETC components shown to generate superoxide anion. Other sources of superoxide can be enzymatic reactions that transfer electrons to the ETC such as mitochondrial glycerol 3-phosphate dehydrogenase (mGPDH), and the last step of β-oxidation, electron-trasferring flavoprotein ubiquinone oxidoreductase (ETFQOR) or dehydrogenases such as pyruvate dehydrogenase (PDH), 2-oxoglutarate dehydrogenase (OGDH) and branched-chain 2-oxoacid dehydrogenase (BCKDH). Superoxide generated in the mitochondrial matrix by these sites is dismutated to hydrogen peroxide by SOD2. Moreover, in response to stress p66Shc translocates to mitochondria to directly stimulate hydrogen peroxide generation by transferring electrons to cytochrome *c*. Hydrogen peroxide is further inactivated using the reducing equivalents of NADPH by mGSH/Gpx or Prx3/Trx2 antioxidant systems, yielding water. Mn-dependent superoxide dismutase 2 (SOD2), GSH peroxidase (GPX1), GSSG-reductase (GR), peroxiredoxin 3 (PRX3), thioredoxin-2 (TRX2), thioredoxin reductase (TrxRD).

Reactive oxygen species can be generated in several intracellular sites, including cytosol, peroxisomes, plasma membrane, and ER. However, mitochondrial ETC is the main cellular process of ROS generation in most cell types in physiological circumstances (Venditti et al., 2013). Although normal electron transport in mitochondria involves four-electron reduction of O_2_ to water, partial reduction reactions occur under physiological conditions, causing release of superoxide anion and hydrogen peroxide (H_2_O_2_). Although ROS can be generated at several sites of the ETC (**Figure 2**; Brand, 2010; Quinlan et al., 2013), complex I and complex III (Venditti et al., 2013) have been shown to be the most important sources of mitochondrial superoxide generation, although significant production of ROS in complex II has recently also been reported (Quinlan et al., 2012).

The primary ROS produced by the ETC is superoxide, a free radical with moderate reactivity, whose generation can lead to more reactive or secondary ROS derivatives. Indeed, superoxide can undergo dismutation to H_2_O_2_, a mild oxidant that can be converted to the highly reactive hydroxyl radical in the presence of transition metals (Fe^2^^+^, Cu^+^) by means of the Fenton reaction. H_2_O_2_ has a longer half-life and can cross membranes (Cadenas and Davies, 2000), consequently it has been identified as a suitable second messenger molecule, in part because of its reactions with specific oxidation-prone protein cysteinyl residues (Sies, 2014), which confers properties to H_2_O_2_ as a mitochondrial signal (Raimundo, 2014).

Reactive oxygen species can attack biomembranes, enzymes, proteins, and nucleic acids (Venditti et al., 2013). These oxidative effects can be neutralized by antioxidant systems, engaging in a delicate balance that determines the fate and impact of ROS in cells. Although oxidative stress was defined originally as a balance between oxidants and antioxidants systems, an equilibrium among antioxidant strategies is needed to avoid the generation of oxidants and ROS (Mari et al., 2010). For instance, if the activity of superoxide scavenging by SOD2 exceeds the capacity to remove the H2O2 generated, this oxidant can cause oxidative damage or be converted to other ROS.

### SUPEROXIDE, HYDROGEN PEROXIDE, AND PEROXYNITRITE GENERATION

Despite the fact that superoxide can be generated in extramitochondrial reactions, in most cell types mitochondria appear as the main source of superoxide generation. From the several sites that can generate superoxide in the mitochondrial matrix, only the superoxide produced at complex III appears to be released both into the matrix and the IMS (Quinlan et al., 2013). This spatial difference (matrix vs. IMS) may determine whether mitochondrial superoxide reaches the cytosol or not. The anionic nature of superoxide and the fact that it is mostly produced in the mitochondrial matrix determine that the bulk of antioxidant defenses to neutralize superoxide and other ROS reside in the matrix. The first line of defense against superoxide is the presence of a specific member of the family of metalloenzymes called superoxide dismutases (SODs), MnSOD or SOD2, specifically located in the mitochondrial matrix, which catalyzes the dismutation of superoxide anion into H_2_O_2_ as shown in **Figure 2**. The dismutation of superoxide can also occur spontaneously, but such reaction is 10^4^ times slower than the enzymatic dismutation by SOD2. The relevance of this enzyme is illustrated by the fact that global SOD2 deficiency leads to neonatal death in mice (Huang et al., 1997). Superoxide released into the IMS can be eliminated by a different SOD isoenzyme (Cu, Zn-SOD, or SOD1), which is found in the cytoplasm of eukaryotic cells, or scavenged by cytochrome *c* plus cytochrome *c* oxidase system (Okado-Matsumoto and Fridovich, 2001). It has been proposed that α-tocopherol can also scavenge superoxide, as suggested by experiments with submitochondrial particles isolated from mice fed with vitamin-E supplemented diet (Chow et al., 1999).

Although the dismutation of superoxide by SOD2 is a predominant source of H_2_O_2_, there are other reactions that directly generate H_2_O_2_ in mitochondria. For example, the redox activitiy of p66Shc within mitochondria has been shown to generate H_2_O_2_ in the absence of superoxide through oxidation of cytochrome *c* (Giorgio et al., 2005). P66Shc normally resides in the cytosol where it is involved in signaling from tyrosine kinases to Ras. However, in response to stress p66Shc translocates to mitochondria to contribute to the generation of H_2_O_2_. Due to the lack of unpaired electrons, H_2_O_2_ is not a free radical but a potent oxidant that can oxidize mitochondrial components (proteins, lipids, DNA). Besides being a potential source of more reactive free radicals via Fenton reaction, physiological generation of H_2_O_2_ fulfills a second messenger role and can be transported across membranes by aquaporins, a family of proteins that act as peroxiporins (Sies, 2014). The detoxification against H_2_O_2_ in mitochondria occurs mainly through the GSH redox system, including the glutathione peroxidases (Gpxs) and GSH reductases, as well as the presence of peroxiredoxins (Prxs; **Figure 2**) using the reducing equivalents of NADPH. Besides these antioxidant defenses that ensure H_2_O_2_ elimination, aquaporins have been shown to modulate mitochondrial ROS generation. In this paradigm, aquaporin 8 silencing, which is specifically expressed in IMM, enhances mitochondrial ROS generation and results in mitochondrial depolarization and cell death (Marchissio et al., 2012). In addition to these conventional sites of mitochondrial ROS generation, it has been recently reported that the branched-chain 2-oxoacid dehydrogenase (BCKDH) complex in mitochondria can produce superoxide and H_2_O_2_ at higher rates than complex I from mitochondria (Quinlan et al., 2014).

Peroxynitrite is a potent oxidant that is generated upon the reaction of superoxide with nitric oxide (NO). Its impact on inactivation of mitochondrial proteins depends on the level of generation in mitochondria. While ETC is the source of superoxide, the existence of mitochondrial NO synthase (mtNOS) that provides the NO required to form peroxynitrite is controversial. Although the existence of mtNOS has been described in mitochondrial fractions from different organs, recent evidence in rat liver mitochondria has questioned the existence of mtNOS, minimizing the contribution of *in situ* NO generation within mitochondrial to the formation of peroxynitrite (Venkatakrishnan et al., 2009). However, since NO is freely diffusible across membranes, it is possible that the mitochondrial production of peroxynitrite may derive from extramitochondrial NO diffusing into mitochondria to react with superoxide generated by ETC.

### GLUTATHIONE REDOX CYCLE

Hydrogen peroxide is rapidly reduced to water mostly by Gpx, which utilizes the reducing equivalents from its substrate GSH. In this enzymatic reaction, GSH becomes oxidized to GSSG, which is recycled back to GSH by the NADPH-dependent GSSG reductase as shown in **Figure 2**. Since GSSG is not readily exported out of mitochondria (Olafsdottir and Reed, 1988; Yin et al., 2012), the activity of GR is an important mechanism to control the level of GSSG in mitochondria. The uncontrolled generation of GSSG during oxidative stress can contribute to mitochondrial dysfunction by glutathionylation of target proteins, as described below. The supply of NADPH is essential to regenerate GSH and dictates the rate of H_2_O_2_ reduction by Gpx while keeping the reduced status of mitochondria.

**FIGURE 3:**
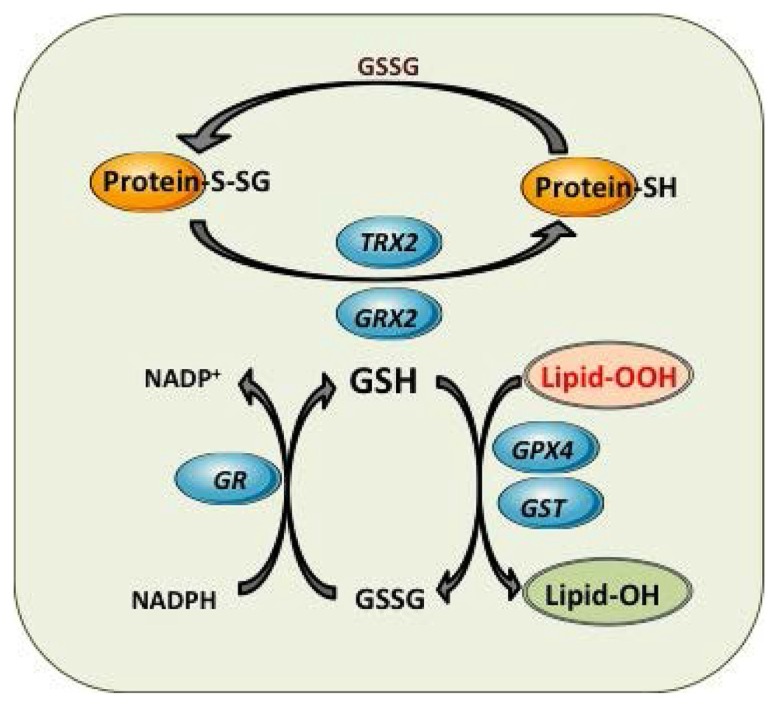
**Mitochondrial GSH redox cycle and interaction with other antioxidant defenses.** Detoxification of harmful lipid peroxides (Lipid-OOH) to their corresponding hydroxides (lipid-OH) by glutathione peroxidase 4 (GPX4) and glutathione-*S*-transferases (GST). Control of mitochondrial protein glutathionylation (Prot-S-SG, Prot -SH) by glutaredoxin (GRX2) and thioredoxin 2 (TRX2).

So far, eight isoforms of Gpx have been identified in humans, which vary in cellular location and substrate specificity (Brigelius-Flohe and Maiorino, 2013). Gpx1 is the major isoform localized in various cellular compartments, including the mitochondrial matrix and IMS (Legault et al., 2000; Mari et al., 2009), which in the liver account for about one third of the total Gpx activity (Chance et al., 1979). This selenium-containing homotetramer protein has substrate specificity for H_2_O_2_ and has been classically believed to be the major H_2_O_2_ reducing enzyme. It also has been described that γ-glutamylcysteine, the intermediate of GSH biosynthesis, is able to act as a Gpx1 cofactor in mitochondrial H_2_O_2_ detoxification, mimicking the physiological properties of GSH (Quintana-Cabrera et al., 2012). Surprisingly, mice with specific genetic deletion of Gpx1 appear phenotypically normal and with normal life span (Ho et al., 1997), suggesting that there are alternative compensatory mechanisms for H_2_O_2_ scavenging in Gpx1 deficiency. However, another report demonstrated mitochondrial stress and bioenergetics defects in GPx1 null mice (Esposito et al., 2000). Besides Gpx1, Gpx4 displays preference for lipid hydroperoxides (**Figure 3**), and hence plays a key role in protecting phospholipids, cholesteryl esters and cardiolipin and defense against apoptosis and maintenance of ETC and OXPHOS (Cole-Ezea et al., 2012). In line with this vital role in mitochondrial defense, Gpx4 null mice die during early embryonic development, while Gpx4^+^^/^^-^ cells are sensitive to oxidative stress triggers (Legault et al., 2000; Muller et al., 2007). Moreover, Gpx4 has been shown recently to modulate ferroptotic cancer cell death, a specific form of cell death characterized by the production of iron-dependent ROS generation (Yang et al., 2014). This process involved metabolic dysfunction that results in increased production of cytosolic and lipid ROS, independently of mitochondria.

### PEROXIREDOXIN–THIOREDOXIN REDOX CYCLE

Peroxiredoxins constitute a family of thiol-specific peroxidases that rely on thioredoxins (Trxs) as the hydrogen donor for the reduction of H_2_O_2_ and lipid hydroperoxides (Chae et al., 1999). Prx3 is the Prx isoform exclusively located in mitochondria, suggesting that it plays a primary line of defense against H_2_O_2_ produced by the mitochondrial respiratory chain. Prx3 homodimer has a redox-sensitive cysteine that upon reaction with H_2_O_2_ is oxidized to Cys-SOH, which then reacts with neighboring Cys-SH of the other subunit to form an intermolecular disulfide that can be readily reduced by thioredoxin reductase 2 (TrxRD2; Chang et al., 2004; Orrenius et al., 2007). The fact that the oxidation state of the active site cysteine of Prx can be transferred to other proteins allows Prx to function as a sensor of H_2_O_2_ (Rhee et al., 2012). Likewise, Prx5, the last identified member of the six mammalian Prxs (Knoops et al., 2011), is widely expressed in tissues but it is not exclusively located in mitochondria. In human cells, it has been shown that Prx5 can be targeted to mitochondria, peroxisomes, cytosol, and nucleus. The targeting of Prx5 to mitochondria is highly conserved among species (Van der Eecken et al., 2011), and it has been associated with the protection of mtDNA from oxidative attacks (Banmeyer et al., 2005). Prx5 is a peroxidase that can use cytosolic or mitochondrial Trx to reduce alkyl hydroperoxides or peroxynitrite with high rate constants, whereas its reaction with H_2_O_2_ is modest (Knoops et al., 2011). Therefore, as opposed to Prx3, Prx5 has been viewed mainly as a cytoprotective antioxidant enzyme rather than as a redox sensor and appears to be a unique Prx exhibiting specific functional and structural feature (Knoops et al., 2011; Zhu et al., 2012).

As noted above, Trxs are responsible for reducing Prx back to their reduced, oxidant-scavenging state, while thioredoxin reductases (TrxRDs) keep the reduced state of Trxs using NAPDH reducing equivalents, as depicted in **Figure 2**. Either Trx and TrxRD are expressed as isoforms for both predominantly cytosolic (Trx1 and TrxRD1) or mitochondrial (Trx2 and TrxRD2) localization (Enoksson et al., 2005). There are direct links between the Trx system and protein glutathionylation (Casagrande et al., 2002); and the direct reduction of mitochondrial glutaredoxin 2 (Grx2) by TrxR is also of important physiological relevance (Johansson et al., 2004; Enoksson et al., 2005). However, the reduction of the intermolecular disulfide of Prx is specific to Trx and cannot be achieved by GSH or Grx (Chang et al., 2004; Orrenius et al., 2007).

Due to their high rate constant and high abundance, Prx are thought to be responsible for scavenging nanomolar concentrations of H_2_O_2_ associated with redox signaling, while Gpx are likely important at higher intracellular concentrations, buffering high ROS levels to avoid cell damage and stress signaling response (Sena and Chandel, 2012). In addition there is emerging evidence indicating that both antioxidant systems (mGSH and Prx3) are mutually regulated. For instance, depletion of mGSH results in Trx2 oxidation (Zhang et al., 2007), while hypercholesterolemic pigs with selective depletion of mGSH in heart mitochondria, exhibit decreased levels of the mitochondria-specific antioxidant enzymes such as SOD2, Trx2, and Prx3 (McCommis et al., 2011). Collectively, these data highlight a key role of mGSH in maintaining a healthy antioxidant system in both systems and on H_2_O_2_ homeostasis.

### DEFENSE AGAINST ELECTROPHILES AND PROTEIN GLUTATHIONYLATION

In addition to the defense against oxidants and ROS, GSH plays also an important role in the protection against electrophiles by glutathione-*S*-transferases (GSTs). Electrophiles can be generated as a consequence of metabolic processes involving both endogenous compounds and xenobiotics. GSTs exhibit a wide intracellular distribution, being localized in mitochondria (GSTA1), cytosol (alpha, mu, pi, and zeta) and membrane-bound (MGST1) isoforms (Aniya and Imaizumi, 2011; Li et al., 2011). Mitochondrial GSTs display both GSH transferase and peroxidase activities that detoxify harmful byproducts through GSH conjugation or GSH-mediated peroxide reduction (**Figure 3**; Hayes et al., 2005; Aniya and Imaizumi, 2011). Among human mitochondrial GSTs, the isoforms hGSTA4-4, hGSTA1, hGSTA2, and hGSTP1 showed peroxidase activity, with hGSTA4-4 exhibiting the highest activity (Gardner and Gallagher, 2001; Gallagher et al., 2006). Moreover, recent studies have shown that GSTA4 expression is selectively downregulated in adipose tissue of obese insulin-resistant C57BL/6J mice and in human obesity-linked insulin resistance (Curtis et al., 2010). Mitochondrial function in adipocytes of lean or obese GSTA4-null mice was significantly compromised compared with wild-type controls and was accompanied by an increase in superoxide anion production.

Glutathionylation, a key mechanism of post-translational modification of proteins, involves the formation of a disulfide bridge between GSH and an available protein cysteine thiol. Non-enzymatic glutathionylation occur mostly during oxidative stress when GSH/GSSG is ~1 and levels of ROS are high. This process is non-specific and can lead to the hyper-glutathionylation of proteins, altering their activity. Enzymatic glutathionylation reactions are tightly controlled and highly specific and are considered a major post-translational modification that occurs in response to fluctuations in local redox environments. The Grx family of proteins plays a key role in the regulation of glutathionylation reactions (**Figure 3**; Lillig et al., 2008). Although Grx are mainly responsible for deglutathionylation reactions, recent evidence has indicated a role for Grx in protein glutathionylation, mediated by the stabilization of a GSH thiyl radical which is then subsequently transferred to an available protein thiol (Starke et al., 2003). In most cases, Grx catalyze the deglutathionylation of proteins GSH disulfide mixtures (PSSGs). Grx exhibit site-specific distribution with Grx1 being specifically located in cytosol while Grx2 localizes in mitochondria. Both Grx catalyze the deglutathionylation of protein targets in two steps; first, the N-terminal cysteine on Grx deglutathionylates PSSG via a thiol disulfide exchange reaction yielding PSH and a Grx–SSG intermediate; second, Grx–SSG binds GSH and the glutathionyl moiety is removed regenerating Grx and producing GSSG. Grx2 has close to 34% homology to Grx1 and was recently identified as the enzyme required for deglutathionylation reactions in mitochondria (Gladyshev et al., 2001; Lundberg et al., 2001; Gallogly et al., 2009; Stroher and Millar, 2012). The catalytic cycle of Grx2 is also quite similar to Grx1 except that the Grx2–SSG intermediate can be reduced by NADPH and TrxRD. It is also important to point out that unlike Grx1, Grx2 complexes iron (Fe), which is required to modulate its activity (Johansson et al., 2004; Lillig et al., 2005). Interestingly, Grx2 has been shown catalyze both the deglutathionylation and glutathionylation of target proteins in mitochondria. The reversible nature of Grx2 is associated with its sensitivity to changes in GSH/GSSG; a high GSH/GSSG promotes protein deglutathionylation and a low GSH/GSSG activates Grx2 glutathionylase activity (Beer et al., 2004; Hurd et al., 2008). The main target for Grx2 in mitochondria is Complex I, although UCP3 and the 2-oxoglutarate dehydrogenase (OGDH) have been also shown to be deglutathionylated by Grx2. The role of Grx2 in maintaining mitochondrial function has been recently shown in heart from Grx2 null mice. Grx2 deletion decreased ATP production by complex I-linked substrates (Mailloux et al., 2014). Grx2^-^^/^^-^ hearts also developed left ventricular hypertrophy and fibrosis and mice developed hypertension.

## MITOCHONDRIA AND CELL DEATH

Besides their fundamental role in energy generation, mitochondria also play a strategic role in the regulation of cell death, including apoptosis (caspase-dependent and independent) and necrosis. Apoptosis describes a programmed mode of cell death that is characterized by a series of biochemical events that ultimately lead to cell fragmentation into compact membrane-enclosed structures, called “apoptotic bodies” that are taken up by neighboring cells and phagocytes, preventing inflammation, and tissue damage (Taylor et al., 2008). Apoptosis is induced via two main routes involving either the mitochondria (the intrinsic pathway) or the activation of death receptors (the extrinsic pathway). Both pathways are linked in some cell types by the cleavage of BID, a proapoptotic member of the Bcl-2 family of proteins, generating tBID in a process catalyzed by caspase-8 activated by the extrinsic pathways (Scaffidi et al., 1998). The intrinsic pathway of apoptosis is activated by stimuli that lead to the permeabilization of the OMM and the subsequent release of proteins from the mitochondrial IMS, such as cytochrome *c* (Martinou and Green, 2001; Kroemer et al., 2007). Cytochrome *c* normally resides within the cristae of the IMM and is sequestered by narrow cristae junctions. As mentioned above, within the IMM, cytochrome *c* participates in the mitochondrial ETC, using its heme group as a redox intermediate to shuttle electrons between complex III and complex IV. However, when the cell detects an apoptotic stimulus, such as DNA damage or metabolic stress, the intrinsic apoptotic pathway is triggered and mitochondrial cytochrome *c* is released into the cytosol (Kroemer et al., 2007). This process is thought to occur in two phases, first the mobilization of cytochrome *c* and then its translocation through permeabilized OMM. In addition to cytochrome *c*, other IMS proteins are also mobilized and released into the cytosol where they promote or counteract caspase activation and hence cell death (Li et al., 2001; Munoz-Pinedo et al., 2006). For instance, the release of Smac/Diablo into the cytosol ensures the efficiency of caspase 3 in proteolyzing target proteins through inhibition of inhibitor of apoptosis proteins (IAPs). Moreover, other specialized mitochondria-residing proteins, such as the apoptosis inducing factor (AIF) and endonuclease G, are translocated to the nuclei following their release from mitochondria and promote peripheral chromatin condensation and high molecular weight DNA fragmentation. While the above evidence indicates that the mitochondrial apoptotic pathway promotes cell death, recent provocative evidence has shown that the intrinsic apoptosis pathway mediates the pro-longevity response to mitochondrial ROS in *Caenorhabditis elegans* by triggering a unique pattern of gene expression that modulates stress sensitivity and promotes survival (Yee et al., 2014). Whether this newly described pathway has implication in mammals needs further verification.

### MITOCHONDRIAL MEMBRANE PERMEABILIZATION AND RELEASE OF PROAPOPTOTIC PROTEINS

While mitochondrial proteins are normally secured in the IMS, understanding the mechanism of release may be of relevance to control cell death. The rupture of the physical barrier (OMM) that limits their release into the cytosol constitutes a point-of-no-return in cell death (Martinou and Green, 2001; Kroemer et al., 2007). Current evidence supports the existence of two compatible mechanisms leading to the breakage of OMM: the mitochondrial permeability transition (MPT), and the permeabilization of OMM without disruption of the inner membrane. The former is a process characterized by mitochondrial swelling, IMM permeabilization and OMM rupture as a secondary event. On the other hand, there is evidence indicating the selective permeabilization of OMM in the absence of disrupted inner membrane. The relative prevalence of these pathways in the regulation of cell death is not definitively established. One important feature of mitochondrial permeabilization is the loss of function resulting in the inability of mitochondria to synthesize ATP through the OXPHOS. However, while the final outcome of mitochondrial dysfunction is cell death, the phenotype of death (apoptosis and/or necrosis) depends on the level of cellular ATP, as ATP is required for the efficient assembly of the apoptosome. Alternatively to MPT in the control of OMM permeabilization, Bcl-2 family members are also known to play a major function. Bcl2-family death agonists induce OMM permeabilization, thereby promoting cytochrome *c* release, whereas Bcl2-family death antagonists prevent it. Thus, Bcl2-family proteins control mitochondrial integrity, regulate cytochrome *c* release and intrinsic apoptosis (Youle and Strasser, 2008). Under non-apoptotic conditions, Bax is inactive and present in the cytosol as a monomer. Following an apoptotic stimulus, Bax is activated and translocates to the mitochondria, where it undergoes a conformational change and inserts into the OMM. Bax oligomerization is associated with the formation of openings in the OMM to allow the release of cytochrome *c* and other IMS proteins into the cytosol, and hence Bax oligomerization is considered a critical regulatory point in cell death (Youle and Strasser, 2008). Further understanding the mechanisms underlying OMM permeabilization may provide novel strategies to regulate cytochrome *c* and control apoptosis.

### REGULATION OF CELL DEATH BY mGSH

As opposed to apoptosis, necrosis is a morphologically distinct form of cell death responsible for irreversible tissue destruction due to bioenergetic failure and oxidative damage. The fundamental difference relative to apoptosis is the rapid loss of cellular membrane potentials due to energy depletion and ion pump/channel failures, leading to swelling, rupture, and cytolysis. MPT is a regulated non-selective water and solute-passing protein complex whose molecular characterization remains elusive. Available evidence suggests a role for voltage-dependent anion channel (VDAC), located in the OMM, and adenine nucleotide translocase (ANT) across the IMM (Kroemer et al., 2007; Baines, 2010) and the translocator protein TSPO [previously called peripheral benzodiazepine receptor (PBR)] as components of MPT. However, liver mitochondria from mice lacking ANT1 and ANT2 can still undergo Ca^2^^+^-induced swelling and MPT, although at a higher threshold, which has been interpreted as evidence against a role for ANT in MPT (Kokoszka et al., 2004). However, recent evidence has demonstrated that TSPO is dispensable for MPT (Sileikyte et al., 2014). In particular, heart mitochondria from mice with selective TSPO deletion in hearts undergo MPT and are as sensitive to ischemia–reperfusion injury as hearts from control mice. In contrast, the prolyl isomerase cyclophilin D in the mitochondrial matrix is an essential regulator of MPT and the only genetically proven indispensable MPT component (Baines et al., 2005; Basso et al., 2005; Nakagawa et al., 2005; Schinzel et al., 2005). Upon oxidative stress, sudden MPT causes massive ion influx that dissipates mitochondrial membrane potential and shuts down OXPHOS, ATP production and ROS overgeneration. Concomitantly, water influx causes matrix swelling, rupture of the rigid OMM and release of apoptogenic proteins sequestered in IMS although, apoptotic cell death under MPT is inhibited due to energetic failure and ATP exhaustion and oxidative stress-mediated caspase inactivation.

A critical step in mitochondrial apoptosis is the mobilization of cytochrome *c* from IMS. It has been proposed that during mobilization cytochrome *c* detaches from the IMM and dissociates from the membrane phospholipid cardiolipin. A significant proportion of the cytochrome *c* in the mitochondria seems to be associated with cardiolipin, involving two major mechanisms. At physiological pH, cytochrome *c* has a net positive charge (+8), establishing an electrostatic bond with the anionic cardiolipin (Gonzalvez and Gottlieb, 2007). In addition, cytochrome *c* has a hydrophobic channel through which one of the four-acyl chains of cardiolipin inserts. The other chains of cardiolipin remain in the membrane, thereby anchoring cytochrome *c* to the IMM. One mechanism that contributes to cytochrome *c* detachment from IMM involves cardiolipin oxidation because oxidized cardiolipin has a much lower affinity for cytochrome *c* than the reduced form. Cardiolipin can be oxidized by ROS or by the cardiolipin–cytochrome *c* complex (Kagan et al., 2005). Detachment of cytochrome *c* from cardiolipin might also be triggered by increased cytosolic calcium, which weakens the electrostatic interaction between cytochrome *c* and cardiolipin and further generates ROS via MPT.

In addition, it has been described that oxidized cardiolipin modulates the biophysical properties of OMM to allow oligomerized Bax to insert and permeabilize the OMM (Mari et al., 2008; Montero et al., 2010; Landeta et al., 2011). Since mitochondrial ROS contribute to cardiolipin oxidation and are controlled by antioxidants (Mari et al., 2008, 2009), mGSH arises as an important modulator of apoptotic cell death by indirectly controlling the redox state of cardiolipin (Mari et al., 2008; Montero et al., 2010). mGSH not only regulates cell death susceptibility but the outcome of cell death (necrosis or apoptosis). Thiol redox status regulate MPT and enhanced ROS generation can target critical cysteine residues in cyclophilin D, implying that mGSH depletion would favor MPT via redox pathways targeting MPT components. In addition, through modulation of cardiolipin redox state, mGSH can also regulate OMM permeabilization via MPT and the release of apoptogenic proteins.

## GLUTATHIONE IMPORT TO MITOCHONDRIA

As indicated above, despite the fact that the concentration of mGSH is high, GSH is not synthesized *de novo* in the mitochondrial matrix, as this organelle lacks the enzymes required for GSH synthesis. Furthermore, GSH has an overall negative charge at physiological pH and mitochondria exhibit a large negative membrane potential. Moreover, although GSH can cross OMM, its transport into mitochondrial matrix cannot be explained by simple diffusion. Therefore, mGSH arises from the cytosol GSH by the activity of specific carriers (**Figure 1**; Griffith and Meister, 1985). Accordingly, recent findings using dynamic oxidant recovery assays and GSH-specific fluorescent reporters, established that free communication of GSH pools exists between cytosol and IMS. In contrast, no appreciable communication was observed between the GSH of the IMS and matrix (Kojer et al., 2012). Based on substrate specificity, potential candidates to transport GSH into mitochondria have been identified, including the 2-oxoglutarate carrier (OGC; SLC25A11) and the dicarboxylate carrier (DIC; SLC25A10; Chen and Lash, 1998; Chen et al., 2000; Coll et al., 2003; Wilkins et al., 2012), mainly in kidney and liver, and tricarboxylate carrier (TTC, SLC25A1) in brain mitochondria and astrocytes (Wadey et al., 2009). OGC imports cytosolic GSH into mitochondria in exchange for 2-oxoglutarate (2-OG) and other dicarboxylates. Instead, DIC mediates electro-neutral exchange of dicarboxylates or GSH for inorganic phosphate (Mari et al., 2009). The relative contribution of each system is different depending on the cell type, as discussed in the following section.

### TISSUE-SPECIFIC FEATURES OF GLUTATHIONE IMPORT TO MITOCHONDRIA

Previous studies in a renal proximal tubular cell line, NRK-52E, indicated that overexpression of OGC and DIC increased mGSH levels and protected against oxidant-mediated cell death (Lash et al., 2002; Xu et al., 2006). Similar findings were recently reported in primary renal proximal tubular cells from uninephrotectomized rats (Benipal and Lash, 2013). The findings with DIC overexpression in kidney cells indicated a role for this carrier in the mitochondrial transport of GSH in exchange with inorganic phosphate. In contrast to kidney, no clear evidence for DIC in the transport of mitochondrial GSH was found in rat liver (Coll et al., 2003). The functional expression in *Xenopus laevis* oocytes microinjected with the DIC cRNA from rat liver did not result in significant GSH transport activity (Coll et al., 2003). Moreover, in contrast to rat kidney mitochondria, the import of GSH in rat liver mitochondria showed both a high affinity and low affinity transport component (Martensson et al., 1990). Likewise, kinetic analyses of 2-oxoglutarate transport in rat liver mitochondria indicated the presence of a single Michaelis–Menten component with kinetic parameters in the range of those reported previously for kidney mitochondria (Chen et al., 2000; Coll et al., 2003). These findings suggest that the OGC accounts for the low-affinity high capacity of GSH transport in liver mitochondria, and imply that the nature of the high affinity GSH transporter remains to be identified. Also, OGC and DIC together accounted for only an apparent 45–50% of the total GSH uptake in liver mitochondria, in contrast to 70–80% described in kidney mitochondria (Zhong et al., 2008).

Interestingly, it has been suggested that Bcl-2 participates as a regulator of mGSH transport by modulating the affinity of OGC for GSH (Wilkins et al., 2012). Bcl-2 and OGC appear to act in a coordinated manner to increase the mGSH pool and to enhance the resistance of neurons to mitochondrial oxidative stress. In line with this outcome, stable motoneuron-like cell lines overexpressing OGC displayed an increased expression of Bcl-2 protein, an effect that was dependent on the mGSH increase. Conversely, a knockdown of Bcl-2 provoked a decrease in mGSH and a concomitant oxidative stress sensitization (Wilkins et al., 2014). Therefore, the antioxidant-like and antiapoptotic function attributed to Bcl-2 could, at least in part, depend on its potential to regulate the mGSH transport and status.

In brain, the properties of GSH transport in isolated rat brain mitochondria seemed to be different from those reported previously for kidney mitochondria, as they were influenced most by inhibitors of the tricarboxylate carrier, citrate, isocitrate, and benzenyl-1,2,3-tricarboxylate (Wadey et al., 2009). Moreover, in mouse brain mitochondria another study showed that OGC and DIC are both expressed in cortical neurons and astrocytes (Kamga et al., 2010). In addition, butylmalonate, an inhibitor of DIC, significantly decreased mGSH, suggesting DIC as the major GSH transporter in mouse cerebral cortical mitochondria (Kamga et al., 2010). It has been shown that pharmacological inhibition or knockdown of a single mGSH transporter significantly sensitized neurons to oxidative and nitrosative stress (Wilkins et al., 2013). Interestingly, a role for UCP2 in the transport of mGSH has been described in neurons, suggesting that the transport of protons back into the matrix by UCP2 may favor the movement of GSH (de Bilbao et al., 2004). These studies suggest that multiple IMM anion transporters might be involved in mGSH transport and that they might differ in different cell populations within the brain. These findings indicate that mGSH levels and its transport are major determinants in brain cell susceptibility to oxidative stress, although little is known about the regulation of the mGSH transport in brain.

### MITOCHONDRIAL MEMBRANE PROPERTIES AND IMPACT ON GLUTATHIONE IMPORT

**FIGURE 4:**
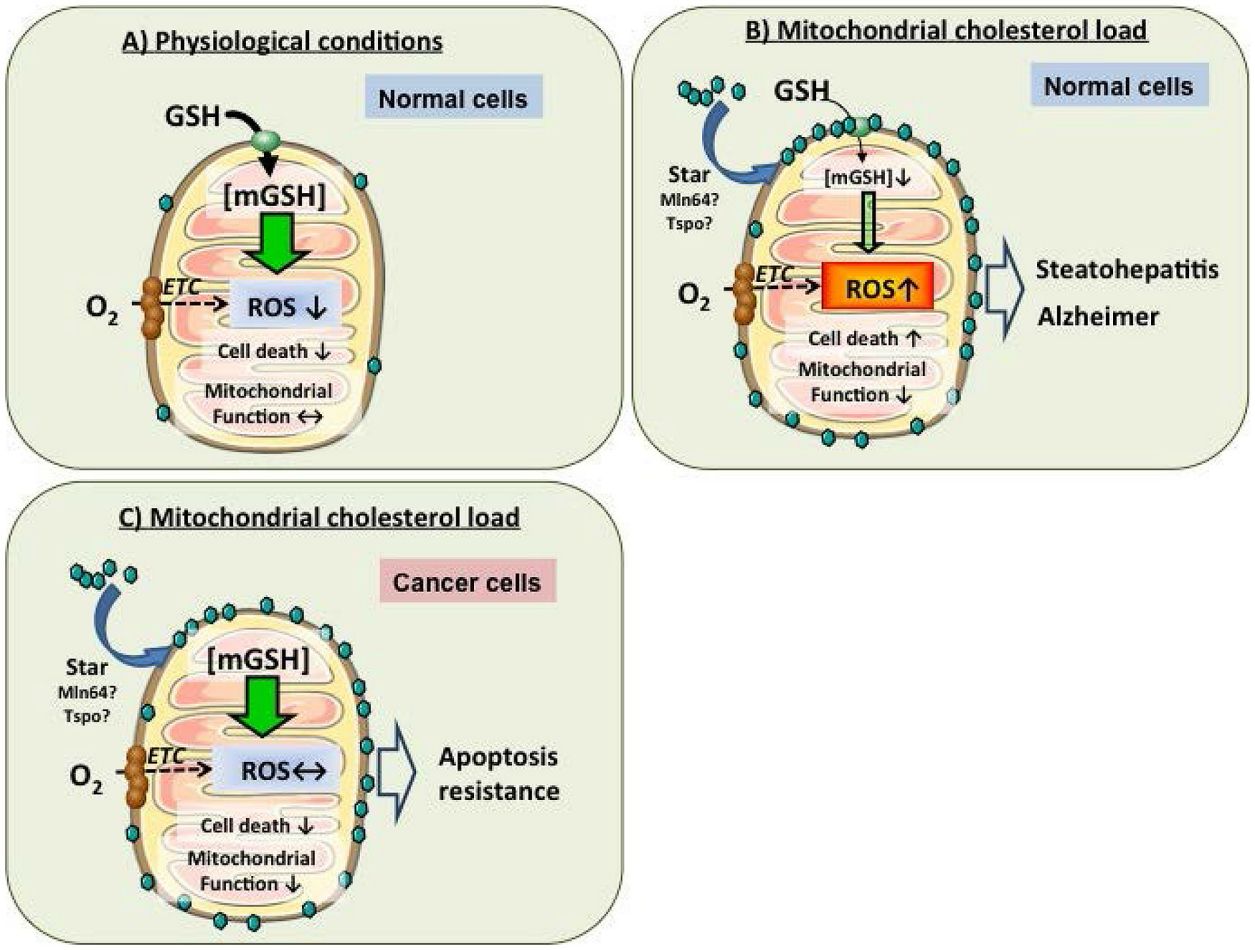
**Regulation of mGSH by mitochondrial cholesterol loading in health and disease.** **(A)** In healthy mitochondria, mGSH levels can cope with the ROS generated in physiological conditions, avoiding cell death induction and maintenance of mitochondrial functions. **(B)** In cholesterol-enriched mitochondria, the mGSH transport is impaired resulting in mGSH depletion. mGSH levels below a certain threshold compromise ROS detoxification, leading to oxidative stress and resulting ultimately in higher susceptibility to cell death. **(C)** However, cancer cells exhibit increased cholesterol enrichment of mitochondrial membrane but paradoxically maintain mGSH levels despite changes in membrane physical properties by an as yet uncharacterized mechanism. Increased mitochondrial cholesterol and mGSH protect against mitochondrial membrane permeabilization and cell death. Thus, targeting mitochondrial cholesterol or mGSH may be a novel approach in the treatment of cancer.

Previous studies in liver mitochondria have revealed that membrane dynamics regulate the transport of mGSH. Membrane physical properties are mainly regulated by fatty acid composition and the cholesterol/phospholipid molar ratio (Coll et al., 2003; Lluis et al., 2003; Ikonen, 2008). Parallel to the findings from rat liver mitochondria, it has been recently reported that mitochondrial cholesterol enrichment, resulting in mGSH depletion, is a major mechanism of anthrax lethal toxin-induced macrophage cell death (Ha et al., 2012). Mitochondria are cholesterol-poor organelles compared to plasma membrane, and this regulated transport of cholesterol in mitochondria plays physiological role in the synthesis of bile acids in liver and steroidogenic hormones in other tissues. (Garcia-Ruiz et al., 2009; Montero et al., 2010). Consistent with the role of cholesterol in the regulation of membrane dynamics, cholesterol loading in mitochondrial membrane results in increased membrane order parameter and in the reduction in the activity of specific membrane carriers, i.e., GSH transport system without effect on other transporters, indicating that the impact of changes in membrane dynamics on carrier function is not universal (Fernandez et al., 2009a; Ha et al., 2012). Moreover, functional expression studies in *X. laevis* oocytes demonstrated that the OGC is sensitive to increased membrane order caused by cholesterol loading (Coll et al., 2003). Thus, cholesterol regulates the transport of mGSH, which in turn, modulates susceptibility to oxidative stress and cell death, therefore emerging as an important target in pathophysiology of diverse diseases such as steatohepatitis (SH) or Alzheimer’s disease (AD; **Figure 4**; Krahenbuhl et al., 1995; Armstrong and Jones, 2002; Garcia-Ruiz et al., 2002; Fernandez-Checa and Kaplowitz, 2005; Lluis et al., 2005, 2007; Mari et al., 2006, 2008, 2009; Lu and Armstrong, 2007; Fernandez et al., 2009b; Fernandez-Checa et al., 2010).

Based on the above findings, understanding the regulation of mitochondrial cholesterol trafficking may be of potential relevance in cell death regulation and disease progression. Given its lipophilic properties and water insolubility, non-vesicular transport by specific carriers stands as the major mechanism of cholesterol transport between organelles. In particular, mitochondrial cholesterol transport is preferentially regulated by the steroidogenic acute regulatory domain 1 (StARD1), the founding member of a family of lipid transporting proteins that contain StAR-related lipid transfer (START) domains (Miller, 2013). StARD1 is an OMM protein which was first described and best characterized in steroidogenic cells where it plays an essential role in cholesterol transfer to the IMM for metabolism by cholesterol side chain cleavage enzyme (CYP11A1) to generate pregnenolone, the precursor of steroids. Pregnenolone synthesis in mitochondria is limited by the availability of cholesterol in the IMM (Clark, 2012). Despite similar properties with StARD1, other StART members cannot replace StARD1, as germline StARD1 deficiency is lethal due to adrenocortical lipoid hyperplasia (Caron et al., 1997). For instance, targeted mutations in MLN64 (StARD3), another START member with wide tissue distribution, have been shown to cause minor alterations in metabolism and intracellular distribution of cholesterol, questioning its contribution to intramitochondrial cholesterol trafficking (Kishida et al., 2004; Miller, 2007). StARD1 activation and regulation is complex and poorly understood. Its activation is regulated at the transcriptional and post-translational levels, as StARD1 phosphorylation at serine194 has been shown to enhance the trafficking of cholesterol to IMM in murine steroidogenic cells, resulting in increased steroidogenesis (Arakane et al., 1997; Kil et al., 2012). Moreover, the role of ER stress in the regulation of StART family members has been limited to StARD5 with conflicting results reported for StARD4. However, recent data have provided evidence that ER stress induces the transcriptional upregulation of StARD1 independently of SREBP regulation (Fernandez et al., 2013). High cholesterol feeding caused the repression of SREBP-2 regulated genes, HMG-CoA reductase, but not that of StARD1. Similar findings have been reported in brain mitochondria in a murine model of AD (Barbero-Camps et al., 2014). Furthermore, the increase in mitochondrial cholesterol in brain mitochondria of AD was not accompanied by a selective increase in mitochondrial-associated membranes (MAMs), corresponding to the contact between ER and mitochondria, suggesting that StARD1-mediated cholesterol trafficking to mitochondria is independent of MAM, a specific membrane domain made of ER and mitochondria bilayers, which is thought to be of relevance in the traffic of lipids. TSPO, a protein particularly abundant in steroidogenic tissues and primarily localized in the OMM, has been suggested to play an important role in steroidogenesis via the transport of cholesterol to the IMM (Papadopoulos and Miller, 2012; Miller, 2013). However, quite interestingly, recent studies using tissue-specific genetic deletion of TSPO demonstrated that TSPO is dispensable for steroidogenesis in Leydig cells (Morohaku et al., 2014), questioning the relevance of previous findings on TSPO using pharmacological ligands and inhibitors. These data underscore that TSPO does not play a significant role in the trafficking of cholesterol to IMM, and highlights the relevance of StARD1 in this process.

Finally, a role for caveolin-1 (CAV1) in mitochondrial cholesterol has been recently reported. CAV1 is a key component of caveolae, specialized membrane domains particularly enriched in cholesterol and sphingolipids, and CAV is known to bind cholesterol with high affinity (Murata et al., 1995; Pol et al., 2005; Boscher and Nabi, 2012). CAV’s ability to move between cell compartments, mitochondria–ER and plasma membrane, might contribute to regulation of cholesterol fluxes and distributions within cells (Pol et al., 2001, 2005; Parton and Simons, 2007; Bosch et al., 2011). In line with these features, CAV1 deficiency has been shown to increase mitochondrial cholesterol in hepatocytes causing perturbations in mitochondrial membrane dynamics and function, and as expected, mGSH depletion (Bosch et al., 2011). The mitochondrial dysfunction sensitizes CAV1 null mice to SH and neurodegeneration. Whether the trafficking of mitochondrial cholesterol in the absence of caveolin-1 occurs via MAM or StARD1 remains to be further investigated.

## ROLE OF MITOCHONDRIA AND MITOCHONDRIAL GSH IN DISEASE

Given the role of mitochondria in oxygen consumption, metabolism and cell death regulation, alterations in mitochondrial function or dysregulation in cell death pathways contribute to many diseases such as cancer, SH, or neurodegeneration. Consistent with its role in regulating mGSH, mitochondrial cholesterol accumulation emerges as a key factor regulating ROS and electrophile detoxification, and hence disease progression by sensitizing to secondary hits such as TNF, hypoxia or toxic amyloid peptides. In the following sections we will briefly cover examples of diseases where mitochondria cholesterol, oxidative stress, and mGSH depletion have been shown to play a role, such as cancer, fatty liver disease, and AD.

### CANCER BIOLOGY AND THERAPEUTICS

Cancer cells exhibit critical metabolic transformations induced by mutations in oncogenes (gain-of-function) and tumor suppressor genes (loss-of-function) that result in cell deregulation associated with enhanced cellular stress. Adaptation to this stress phenotype is required for cancer cells to survive and involves the participation of genes that regulate generation and sensitization to ROS-mediated cell death. In this context, small molecules that selectively kill cancer cells are a promising approach for the treatment of cancer. Experiments using a cell-based small-molecule screening and quantitative proteomics, revealed the potential of piperlongumine, a natural product isolated from the plant species *Piper longum L*, as a cytotoxic agent triggering apoptosis and necrosis in leukemia cells (Bezerra et al., 2007). Moreover, piperlongumine induces ROS generation resulting in the killing of transformed cells *in vitro* and *in vivo* but not primary normal cells (Raj et al., 2011). Piperlongumine leads to decreased GSH and increased GSSG levels in cancer cells without effects in non-transformed cells, and these effects paralleled the ability of piperlongumine to cause alterations in mitochondrial morphology and function. Consequently, co-treatment with piperlongumine and *N*-acetyl-L-cysteine (NAC) prevented piperlongumine-mediated GSH depletion and cell death in cancer cells. These findings support the concept that normal cells have low basal levels of ROS and a diminished reliance on the ROS stress-response, while cancer cells have high levels of ROS, and hence, are expected to have a strong reliance on the ROS stress-response pathway. In line with the relationship between ROS and cancer it has been suggested that antioxidants may protect against cancer. However, randomized clinical trials have produced inconsistent results and some studies indicated that antioxidants increase cancer risk (van Zandwijk et al., 2000; Klein et al., 2011; Watson, 2013). A recent study in oncogene-induced lung cancer demonstrated that treatment with NAC and vitamin E accelerate cancer progression, stimulating cell proliferation by reducing ROS, DNA damage, and p53 expression (Sayin et al., 2014). The use of small molecules that alter the levels of ROS such as β-phenethyl isothiocyanate (PEITC), buthionine sulphoximine, curcumin, or 2-cyano-3,12-dioxooleana-1,9-diene-28-oic acid (CDDO) derivatives, has been suggested for the treatment of cancer by promoting ROS generation and GSH depletion in cancer cells (Schumacker, 2006; Trachootham et al., 2006; Yue et al., 2006; Ravindran et al., 2009). Interestingly, mGSH depletion has also been associated with apoptosis or autophagy induced by chemotherapeutic drugs. For instance, the novel triterpenoid methyl CDDO derivative (CDDO-Me) potently induced cytotoxicity in imatinib-resistant myeloid leukemia cells, accompanied by a rapid and selective depletion of mGSH resulting in increased generation of ROS and mitochondrial dysfunction (Samudio et al., 2005, 2008). Moreover, PEITC caused a rapid depletion of mGSH and a significant elevation of ROS and NO, induced a disruption of the mitochondrial electron transport complex I, and a significant suppression of mitochondrial respiration that resulted in cytotoxicity in leukemia cells (Chen et al., 2011).

As mGSH is regulated by cholesterol, as described above, the trafficking of mitochondrial cholesterol may modulate cancer cell biology. Cholesterol metabolism is deregulated in tumors, which exhibit a paradoxical stimulation in *de novo* cholesterol synthesis despite hypoxia-mediated downregulation of HMG-CoA reductase by hypoxia (Nguyen et al., 2007; Garcia-Ruiz et al., 2009). In addition to its continued synthesis, cholesterol trafficking to mitochondria has been reported in tumor cells, including in mitochondria from hepatocellular carcinoma (HCC) due to overexpression of StARD1 (Montero et al., 2008). Mitochondrial cholesterol loading in cancer cells may actually account for the recognized mitochondrial dysfunction and resistance to Bax-mediated cell death induced by chemotherapy agents. In line with this hypothesis, treatments that resulted in mitochondrial cholesterol loading in tumor cells impaired stress-induced apoptosis (Lucken-Ardjomande et al., 2008; Montero et al., 2008), while StARD1 knockdown or treatments that resulted in downregulation of cholesterol loading sensitized HCC cells to chemotherapy. These findings identify the mitochondrial cholesterol loading in cancer cells, particularly HCC, as a mechanism contributing to chemotherapy resistance and evasion of Bax-mediated apoptosis (**Figure 4**). While mitochondrial cholesterol depletes mGSH due to impaired transport via OGC in primary hepatocytes and in SH, cancer cells paradoxically maintain mGSH homeostasis by a still ill-defined mechanism that is under investigation.

### ALCOHOLIC AND NON-ALCOHOLIC FATTY LIVER DISEASE

Fatty liver disease represents a spectrum of liver disorders that begins with simple steatosis. This intial stage can progress to SH and culminate in cirrhosis and liver cancer. SH is an intermediate stage of fatty liver disease and one of the most common causes of chronic liver disease worldwide that may progress to cirrhosis and liver cancer. SH is characterized by steatosis, oxidative stress, hepatocellular death, inflammation and fibrosis and encompasses alcoholic (ASH) and non-alcoholic steatohepatitis (NASH). Unfortunately, there is no approved therapy for ASH/NASH, which reflects our incomplete understanding of the underlying mechanisms (Tilg and Diehl, 2000; Angulo and Lindor, 2002; Brunt, 2004). The development of steatosis in ASH/NASH is secondary to the metabolic disturbances in ASH and NASH, including insulin resistance, adipose tissue lipolysis, stimulation of *de novo* lipid synthesis and impaired mitochondrial fatty acid oxidation (Garcia-Ruiz and Fernandez-Checa, 2006; Garcia-Ruiz et al., 2011, 2013a,b). A key concept in SH pathogenesis is the two-hit hypothesis, which posits that hepatic steatosis sensitizes fatty liver to secondary hits, such as inflammatory cytokines and oxidative stress. However, recent evidence has shown that the type rather than the amount of fat plays a critical role in the transition from steatosis to ASH/NASH. In line with this hypothesis, previous studies have shown that chronic alcohol feeding in various models results in the depletion of mGSH due to cholesterol loading in mitochondria (Garcia-Ruiz et al., 1994; Colell et al., 1997, 1998, 2001; Zhao et al., 2002; Garcia-Ruiz and Fernandez-Checa, 2006) and that strategies aimed to correct the loss of mitochondrial membrane fluidity restore the mitochondrial transport of GSH and replenish the mGSH pool in alcohol-fed models. Moreover, recent evidence in rats fed an ethanol-polyunsaturated fatty acid treatment confirmed the mitochondrial cholesterol accumulation and GSH depletion, leading to SH, and these effects were prevented by betaine treatment (Varatharajalu et al., 2014). Altered alcohol-induced ER stress involves alterations in the methionine cycle and hyperhomocysteinemia, and treatment with betaine prevents alcohol-induced ER stress, steatosis, and liver injury (Ji and Kaplowitz, 2003, 2004). Moreover, tauroursodeoxycholic acid, a chemical chaperone shown to prevent ER stress (Ozcan et al., 2006), restored the mGSH pool in alcohol fed rats (Colell et al., 2001) and blocked alcohol-induced ER stress (Fernandez et al., 2013). The mechanisms of alcohol-induced mitochondrial cholesterol trafficking, mediated by alcohol-induced upregulation of StARD1, requires alcohol-induced acid sphingomyelinase activation (Fernandez et al., 2013).

Increased cholesterol synthesis and levels have been reported in liver biopsies from patients with NASH (Puri et al., 2007; Caballero et al., 2009) and mGSH depletion has been observed in models and patients with NASH (Serviddio et al., 2008). This outcome is consistent with the increased expression of StARD1 in patients with NASH but not with simple steatosis (Caballero et al., 2009). In line with these findings, recent data reported that the inhibition of microsomal triglyceride transfer protein, a model of liver steatosis, induced the increase in free cholesterol in mitochondria resulting in mGSH depletion (Josekutty et al., 2013). In addition to mGSH depletion, NASH is also characterized by impaired SOD2 activity, which may contribute the increased generation of mitochondrial superoxide and subsequent peroxynitrite levels that target mitochondrial proteins causing their inactivation. In principle, strategies such as SOD mimetics aimed to improve SOD2 activity may be of relevance in NASH. However, the use of SOD mimetics in parallel with the reported mGSH depletion can cause increased H_2_O_2_ and overall oxidant-dependent liver injury (Montfort et al., 2012). This scenario implies that the combination of SOD mimetics and mGSH replenishment may more efficient in NASH treatment.

### ALZHEIMER DISEASE

Alzheimer disease is a major neurodegenerative disorder and the main cause of adult dementia. The main risk factor for AD is aging and therefore the number of people worldwide facing AD development increases every year. AD is characterized by progressive memory loss, cognitive impairment and disruption of synaptic plasticity. Although there are recommended therapies for AD, such as acetylcholinesterase inhibitors and the *N*-methyl-D-aspartate receptor antagonists, they are inefficient and do not prevent disease progression, reflecting our incomplete understanding of AD pathogenesis. Experimental models and human data established two main theories underlying AD, the accumulation of toxic amyloid β (Aβ) peptides, characteristic of senile plaques, and the aggregation of tau protein, a microtubule-associated protein expressed in neurons that is involved in the stabilization of microtubules in the cytoskeleton. The pathogenic processing of the amyloid precursor protein (APP) leads to toxic Aβ generation and is considered a critical mechanism of AD. Accordingly, a coding mutation (A673T) in APP has been recently shown to protect against AD and age-related cognitive decline in elderly Icelanders (Jonsson et al., 2012). This substitution, which is close to the aspartyl protease β-site in APP, reduces the formation of amyloidogenic peptides *in vitro* by 40%. The protective effect of the A673T substitution against AD provides strong evidence for the hypothesis that reducing the β-cleavage of APP may protect against the disease. Amiloidogenic processing of APP yields toxic Aβ peptides. In this pathway, the β- and γ-secretases cleave APP at the N- and C-termini of the Aβ peptide, respectively. β-Secretase has been characterized as a membrane-bound aspartic protease termed beta-site APP-cleaving enzyme 1 (BACE1), while γ-secretase is a complex comprised of presenilin-1 or -2, nicastrin, anterior pharynx-defective 1 (Aph-1) and presenilin enhancer 2 (Pen-2; Haass, 2004). Another novel member of the γ-secretase complex has been identified. β-arrestin 2 physically associates with the Aph-1α subunit of the γ-secretase complex and redistributes the complex toward detergent-resistant membranes, increasing the catalytic activity of the complex (Thathiah et al., 2013). Moreover, β-arrestin 2 expression is elevated in individuals with AD and its overexpression leads to an increase in Aβ peptide generation, whereas genetic silencing of Arrb2 (encoding β-arrestin 2) reduces generation of Aβ in cell cultures and in Arrb2^-^^/^^-^ mice. In addition to its amyloidogenic processing by β- and γ-secretases, APP can be cleaved within the Aβ domain by α-secretase. This non-amyloidogenic processing prevents the deposition of intact Aβ peptide and results in the release of a large soluble ectodomain, sAPPα, from the cell, which has neuroprotective and memory-enhancing effects. Members of the ADAMs, a disintegrin and metalloprotease family of proteases, have been shown to possess α-secretase activity (Hooper and Turner, 2002). The pathogenic processing of APP into toxic Aβ fragments occurs in cholesterol-enriched membrane domains of the plasma membrane, known as lipid rafts, consistent with the recognized role of cholesterol in AD pathogenesis based upon experimental and epidemiological evidence linking plasma cholesterol levels and AD development (Notkola et al., 1998; Wolozin et al., 2000; Anstey et al., 2008). High cholesterol levels correlated with Aβ deposition and the risk of developing AD, while patients taking the cholesterol-lowering drug statins were found to have a lower incidence of the disease (Notkola et al., 1998; Wolozin et al., 2000). Exploiting the relative detergent insolubility of lipid rafts, there has been evidence indicating the localization of APP, the α-, β- and γ-secretases in rafts (Wahrle et al., 2002; Vetrivel et al., 2005). In addition, the activities of BACE1 and γ-secretase are stimulated by lipid components of rafts, such as glycosphingolipids and cholesterol (Sawamura et al., 2004; Kalvodova et al., 2005; Ariga et al., 2008; Osenkowski et al., 2008). Besides its extracellular deposition, current evidence indicates the processing and targeting of APP and Aβ to intracellular sites, including mitochondria (Lin and Beal, 2006). Moreover, levels of mitochondrial APP are higher in affected brain areas and in subjects with advanced disease symptons (Devi et al., 2006). Immunoelectron microscopy analyses indicated the association of APP with mitochondrial protein translocation components, TOM40 and TIM23, which correlated with decreased import of respiratory chain subunits *in vitro*, decreased cytochrome oxidase activity, increased ROS generation and impaired mitochondrial reducing capacity (Devi et al., 2006). Although the molecular mechanisms of mitochondrial Aβ targeting remains poorly understood, Aβ stimulates mitochondrial ROS generation, contributing to Aβ toxicity in neurons (Behl et al., 1994; Casley et al., 2002; Lustbader et al., 2004). In addition to the amyloidogenic effect of cholesterol by fostering Aβ generation from APP, recent data has provided evidence that mitochondrial cholesterol accumulation sensitizes neurons to Aβ-induced neuroinflammation and neurotoxicity by depleting mGSH, effects that are prevented by mGSH replenishment (Fernandez et al., 2009b). The mechanism of mitochondrial cholesterol accumulation involves the upregulation of StARD1 induced by Aβ *via* ER stress (Barbero-Camps et al., 2014), confirming previous findings in hepatocytes (Fernandez et al., 2013). Although not reported in patients, the trafficking of cholesterol to mitochondria may be of clinical relevance to human AD due to the described enhanced expression of StARD1 in pyramidal hippocampal neurons of AD-affected patients (Webber et al., 2006). Moreover, a novel mouse model engineered to have enhanced cholesterol synthesis by SREBP-2 overexpression superimposed to APP/PS1 mutations triggered Aβ accumulation and tau pathology (Barbero-Camps et al., 2013). This triple transgenic model exhibited increased mitochondrial cholesterol loading and mGSH depletion and accelerated Aβ generation by β-secretase activation compared to APP/PS1 mice (Barbero-Camps et al., 2013). Moreover, SREBP-2/APP/PS1 mice displayed synaptotoxicity, cognitive decline, tau hyperphosphorylation and neurofibrillary tangle formation in the absence of mutated tau, indicating that cholesterol, particularly mitochondrial cholesterol, can precipitate Aβ accumulation and tau pathology. Importantly, *in vivo* replenishment of mGSH with cell- permeable GSH monoethyl ester (GSH–EE) attenuated neuropathological features of AD in SREBP-2/APP/PS1 mice.

In addition to the proteolytic processing by secretases, APP and its corresponding C-terminal fragments are also metabolized by lysosomal proteases. SORLA/SORL1 is a unique neuronal sorting receptor for APP that has been causally implicated in sporadic and autosomal dominant familial AD. Brain concentrations of SORLA are inversely correlated with Aβ in mouse models and AD patients. Indeed, transgenic mice overexpressing SORLA exhibit decreased Aβ concentrations in brain (Caglayan et al., 2014). Mechanistically, Aβ binds to the amino-terminal VP10P domain of SORLA and this binding is impaired by a familial AD mutation in SORL1. Moreover, sphingosine-1-phosphate (S1P) accumulation by S1P lyase deficiency has recently been shown to impair lysosomal APP metabolism, resulting in increased Aβ accumulation (Karaca et al., 2014). The intracellular accumulation of S1P interferes with the maturation of cathepsin D and degradation of Lamp2, suggesting a general impairment of lysosomal function and autophagy. As sphingolipids have strong affinity to bind cholesterol (Slotte, 1999; Ridgway, 2000), it is conceivable that increased lysosomal cholesterol may contribute to impaired lysosomal Aβ degradation in the S1P lyase knockout mice. However, this aspect remains to be investigated, raising the question of whether lysosomal cholesterol plays a role in lysosomal Aβ degradation and hence has any relevance in AD. Quite intriguingly, recent findings have reported increased expression of the lysosomal cholesterol transporter Niemann–Pick type C disease 1 (NPC1) in AD (Kagedal et al., 2010). NPC is an endolysosomal protein essential for the intracellular regulation of cholesterol and its mutation and loss-of-function elicits the lysosomal storage disease NPC disease, characterized by the accumulation of lysosomal cholesterol and sphingolipids. NPC1 expression was described to be upregulated at both mRNA and protein levels in the hippocampus and frontal cortex of AD patients compared to controls subjects. However, no difference in NPC1 expression was detected in the cerebellum, a brain region that is relatively spared in AD. Moreover, murine NPC1 mRNA levels increased in the hippocampus of 12-month-old APP/PS1 mice compared to wild-type mice. These findings strongly suggest the lack of lysosomal cholesterol accumulation in AD, and imply that lysosomal impairment and subsequent contribution to decreased Aβ degradation in AD may occur through mechanisms independent of cholesterol accumulation in lysosomes. Although several similarities exist between NPC disease and AD, including altered intracelular cholesterol homeostasis, changes in the lysosomal function, neurofibrillary tangles, and increased Aβ generation and neurodegeneration, the likely common nexus between these diseases is mitochondrial cholesterol loading, rather than lysosomal cholesterol accumulation, as reported both in AD and NPC disease (Yu et al., 2005; Colell et al., 2009; Fernandez et al., 2009b). Thus, targeting mitochondrial cholesterol may be of relevance not only for AD but also for other neurodegenerative and lysosomal storage diseases, including NPC.

## CONCLUSION AND FUTURE APPROACHES

Mitochondria play an essential role in providing the energy needed for multiple signaling cascades and cellular functions. The consumption of molecular oxygen in the respiratory chain not only is the driving force for the ATP synthesis required for cell viability, but also the source of ROS that target mitochondrial and extramitochondrial targets. As described above, mitochondrial oxidative stress and the mGSH depletion are central events of many pathological conditions. However, a challenge to counteract mitochondrial oxidative stress is to recover mGSH pool when GSH transport is defective due to alterations in membrane dynamics triggered by increased mitochondrial cholesterol accumulation. In addition to the ability of mitochondrial-permeable GSH-EE to directly increase mGSH levels bypassing the mitochondrial transport defect, it has been recently described additional strategies that supply mitochondria with GSH, including parental molecules that generate GSH once inside the mitochondrial matrix. This approach has been recently illustrated with the use of *S*-D-lactoylglutathione (Armeni et al., 2014). This compound is an intermediate of the glyoxalase system, which is hydrolyzed in the mitochondrial matrix yielding lactate and GSH; hence showing the ability to replenish mGSH resulting in recovery of mitochondrial function and antioxidant defense. Unlike these permeable GSH prodrugs that directly boost mGSH, strategies aimed to increase cytosol GSH (e.g., NAC) may not be an optimal approach for boosting mGSH and therefore for treatment of SH or AD, as it would result in mainly increasing cytosol GSH without replenishing mGSH levels. Another strategy to combat ROS generation would be the supply of antioxidants that are targeted selectively to mitochondria. Since lipophilic cations accumulate in mitochondria, the covalent attachment of a neutral bioactive compound to a lipophilic cation should lead to its selective delivery to mitochondria. In this regard, alkyl-triphenylphosphonium (TPP) cations are excellent tools for the delivery of compounds to mitochondria as they preferentially accumulate quite efficiently within mitochondria in cells, making it possible to deliver a wide range of mitochondria-targeted lipophilic TPP-labeled cations (Ross et al., 2005; Sheu et al., 2006). This approach has been exploited recently in several contexts by the development of a series of cationic antioxidants targeted to mitochondria, including derivatives of the endogenous antioxidants ubiquinol (MitoQ), alpha-tocopherol (MitoVit E), and of the synthetic spin trap PBN (MitoPBN; Ross et al., 2005). These compounds have been found to block oxidative damage in isolated mitochondria and cells more effectively than untargeted antioxidant analogs due to their concentration within mitochondria. More importantly, oral administration of these compounds leads to their accumulation in the brain, heart, muscle and liver mitochondria (Ross et al., 2005). In fact, MitoQ has been used in a range of *in vivo* studies, in rats and mice, and in two phase II human trials demonstrating that it can be safely delivered to patients with promising results, lending further support that mitochondria-targeted antioxidants may be applicable to a wide range of human pathologies that involve mitochondrial oxidative damage (Smith and Murphy, 2010).

## Conflict of Interest Statement

The authors declare that the research was conducted in the absence of any commercial or financial relationships that could be construed as a potential conflict of interest.

## ABBREVIATIONS

AD: Alzheimer disease
APP: amyloid precursor protein
ASH: alcoholic steatohepatitis
ETC: electron transport chain
Gpx: glutathione peroxidase
Grx: glutaredoxin
GSH: glutathione
GSSG: oxidized glutathione
GST: glutathione-*S*-transferase
IMM: inner mitochondrial membrane
mGSH: mitochondrial glutathione
MPT: mitochondrial permeability transition
NASH: non-alcoholic steatohepatitis
NO: nitric oxide
NPC: Niemann–Pick type C disease
OMM: outer mitochondrial membrane
OXPHOS: oxidative phosphorylation
Prx: peroxiredoxin
ROS: reactive oxygen species
SOD: superoxide dismutase
StARD1: steroidogenic acute regulatory domain 1
Trx: thioredoxin.
